# Managing distance and covariate information with point-based clustering

**DOI:** 10.1186/s12874-016-0218-z

**Published:** 2016-09-01

**Authors:** Peter A. Whigham, Brandon de Graaf, Rashmi Srivastava, Paul Glue

**Affiliations:** 1 Information Science Department, University of Otago, Dunedin, New Zealand; 2 University of Otago, Dunedin, New Zealand; 3 University of Otago, Invercargill, New Zealand

**Keywords:** Deliberate self-harm, Clustering, Ripley’s K, Deprivation, Social contagion, Monte-Carlo simulation, Minkowski distance

## Abstract

**Background:**

Geographic perspectives of disease and the human condition often involve point-based observations and questions of clustering or dispersion within a spatial context. These problems involve a finite set of point observations and are constrained by a larger, but finite, set of locations where the observations could occur. Developing a rigorous method for pattern analysis in this context requires handling spatial covariates, a method for constrained finite spatial clustering, and addressing bias in geographic distance measures. An approach, based on Ripley’s K and applied to the problem of clustering with deliberate self-harm (DSH), is presented.

**Methods:**

Point-based Monte-Carlo simulation of Ripley’s K, accounting for socio-economic deprivation and sources of distance measurement bias, was developed to estimate clustering of DSH at a range of spatial scales. A rotated Minkowski L_1_ distance metric allowed variation in physical distance and clustering to be assessed. Self-harm data was derived from an audit of 2 years’ emergency hospital presentations (*n* = 136) in a New Zealand town (population ~50,000). Study area was defined by residential (housing) land parcels representing a finite set of possible point addresses.

**Results:**

Area-based deprivation was spatially correlated. Accounting for deprivation and distance bias showed evidence for clustering of DSH for spatial scales up to 500 m with a one-sided 95 % CI, suggesting that social contagion may be present for this urban cohort.

**Conclusions:**

Many problems involve finite locations in geographic space that require estimates of distance-based clustering at many scales. A Monte-Carlo approach to Ripley’s K, incorporating covariates and models for distance bias, are crucial when assessing health-related clustering. The case study showed that social network structure defined at the neighbourhood level may account for aspects of neighbourhood clustering of DSH. Accounting for covariate measures that exhibit spatial clustering, such as deprivation, are crucial when assessing point-based clustering.

## Background

Point pattern analysis to assess clustering or dispersion of a set of events in a bounded spatial region is commonly based on quadrant-based sampling aggregations or point-based measures such as the empty space function, pairwise and nearest neighbour distance [1–3]. This work extends the pairwise distance measure of Ripley’s K [2, 4, 5] to assess clustering over a range of spatial scales, while taking into account covariate and metric bias [3, 6]. Ripley’s K function [4] was originally designed for characterising stationary point-patterns for a homogeneous Poisson process. The K function is a cumulative function defined over a range of pairwise distance counts that can distinguish clustered, random and dispersed spatial point patterns as a comparison against complete spatial randomness (CSR). Theoretical comparisons against CSR require an estimate of the intensity of points within a study region. The example presented in this paper evaluates clustering of episodes of deliberate self-harm (DSH) over 2 years in an urban environment. Because the study contains a finite set of points representing residential addresses means that the distance measure may not be planar and placement of points in the study area are not continuous. Similar difficulties have been addressed with modelling point distributions on a network [7, 8], although in this case distance was well defined by network connectivity. The approach presented here also addresses similar issues to the second-order analysis of clustering for inhomogeneous populations [5], where a set of control cases are randomly selected to form a comparative K estimate. However this approach does not consider the influence of clustering due to covariate relationships in the observed point pattern. The spatial variation of disease presents similar issues, but is normally handled by kernel-based regression methods [6], without consideration of the influence of metrics on observed clustering.

Here we present a method to examine clustering for a finite set of point locations and present a method to examine uncertainty in the planar distance measurement. In addition, the observed point data is correlated with a spatial variable that is clustered, and therefore must be accounted for when assessing clustering via an estimate of Ripley’s K.

There has been a long history examining the relationship between social behaviour and the patterning of societal structure [9–11]. Two main theories are generally proposed [11]: behaviour is characterised by the underlying structure of the environment that defines the living conditions of individuals; or that behaviour is influenced by social interaction (often described as social contagion) that results in behaviours being shared and amplified between individuals. Both theories have been used to explain patterns of behavioural clustering and it is generally acknowledged that one cannot occur without the other. For example, Baller and Richardson [11] examined the patterning of suicide within the historical context of French departments from 1872 to 1876, and data for U.S. Counties from 1990. Using area-based spatial analysis methods they concluded that the French example showed clustering after social integration was accounted for in the data, while the U.S. example did not show any residual clustering once social integration was incorporated in the model. They concluded that both concepts of social structure and contagion through imitation were responsible for the spatial patterning of suicide.

Previous clustering methods for self-harm behaviour used area-based counts for index events which aligned with other area-based covariates (such as social deprivation). This allowed regression approaches to be used to account for covariates and spatial lag [12]. Moran’s I or other simple count-based methods were then used to assess clustering [9, 10, 13, 14]. However, the ability to now collect and manage point-based data and incorporate this directly into spatial analysis means there is a need to develop appropriate clustering measures that handle covariate measures with points and address distance bias when Euclidean distance may not be appropriate, or where a distance metric is difficult to define.

## Methods

The second order moment (Ripley’s K) for an unlabelled, homogeneous, isotropic point process observed as a set of points x_i_ ∈ ℝ^2^ is defined as [5]:

$$ \mathrm{K}\left(\mathrm{r}\right)={\uplambda}^{-1}\mathrm{E}\left[{\mathrm{number}\ \mathrm{of}\ \mathrm{other}\ \mathrm{point}\mathrm{s}\ \mathrm{in}\ \mathrm{the}\ \mathrm{process}\ \mathrm{within}\ \mathrm{distance}\ \mathrm{r}\ \mathrm{of}\ \mathrm{a}\ \mathrm{point}\ \mathrm{from}\ \mathrm{x}}_{\mathrm{i}}\right] $$

where λ is the intensity of the point process per unit area. For an isotropic process comparisons with K(r) are normally based on the homogeneous Poisson process Kpois(r) = πr^2^ [2]. For this type of process λ is approximated as the number of points/observed region area. For our derivation of K(r) observations are constrained to a finite set of possible locations. Hence λ is set to the number of points/(maximum observed Euclidean distance between any two points in x_i_).

Consider a set of n possible point locations in a finite region of the plane W. Note that W is not explicitly used; however the locations are bounded. This unordered set of points may be defined as:

$$ \mathbf{x}=\left\{{\mathrm{x}}_1, \dots {\mathrm{x}}_{\mathrm{n}}\right\}\ {\mathrm{x}}_{\mathrm{i}}\in \mathrm{W},\mathrm{n}\ge 0 $$

Each point x_i_ has an associated mark from a finite set of marks M, defining a marked point pattern:

$$ \mathbf{y}=\left\{\left({\mathrm{x}}_1,{\mathrm{m}}_1\right),\dots .,\left({\mathrm{x}}_{\mathrm{n}},{\mathrm{m}}_{\mathrm{n}}\right)\right\},\ {\mathrm{x}}_{\mathrm{i}}\in \mathrm{W},{\mathrm{m}}_{\mathrm{i}}\in \mathrm{M} $$

We observe a set of q marked points **q** ∈ **y**, where q < < n and want to determine if the set **q** deviates from complete spatial randomness. In addition, since the marked pattern may be spatially correlated to the process generating the point pattern, the distribution of the observed marks of **q** must be taken into account when simulating a random sample from **y**. Initially (since q is fixed), we construct the discrete cumulative distribution function for the **q** marks as:

$$ F(X) = {\displaystyle \sum_{k=1}^q} \Pr \left[X={m}_k\right] $$

K(r) is now defined over a set of distance thresholds r_i_ ∈ ℝ for one Monte Carlo simulation as follows:

For each distance threshold r_i_,

1. *P* = {}
2. Repeat until q points have been selected:
  2.1 Draw a uniform random number ρ ∈ [0,1).
  2.2 Determine the mark m_k_ for F(ρ). This corresponds to a proportional selection of a mark value from the frequency distribution of marks for the observed pattern.
  2.3 Select the subset of points **t** = {(x_i_, m_i_) ∈ **y** : m_i_ = m_k_} that correspond to this mark.
  2.4 Randomly select a point s ∈ **t**
  2.5 *P* = P ⋃ s
3. The number of points from the set of points P within the Minkowski Distance L_2_ (Euclidean distance) r_i_ is defined as K(r_i_) = λ^− 1^P.

This method does not assume that the observed marks are clustered, but takes into account their spatial structure when determining K(r). For our case study we show the effect of taking socio-economic structure (defined as a deprivation index) into account has a significant effect on the estimate of clustering (see Results section).

Multiple simulation runs allow an envelope to be constructed. For a one-sided 5 % significant level for q observed points the above simulation is performed 1000 times to define a reference set $\widehat{K}\left({r}_i\right)$. For each distance r_i_ the $\widehat{K}\left({r}_i\right)$ are sorted. A 5 % significance level for the clustering of observed K(r_i_) means that K(r_i_) is greater than the 951st observed value of K from the reference set [7, 15].

### Addressing distance bias

The use of L_2_ distance on the plane (step 3 above) assumes a barrier free, isotropic measure for the distance between points. From a social contagion perspective it is difficult to know what, if any, planar distance is appropriate for the connection between any two index events. In addition, social media and other forms of communication mean that a spatial distance may not be appropriate. Since Ripley’s K requires a distance measure, we would like to confirm that L_2_ distance does not significantly influencing the results.

Consider the Minkowski distance L_1_ (Manhattan or rectilinear distance) defined between two points a(x_1_, y_1_) and b(*x*_2_, y_2_):

$$ {\mathrm{L}}_1\left(\mathrm{a},\mathrm{b}\right)=\left(\left|{\mathrm{x}}_1\hbox{--}\ {\mathrm{x}}_2\right|+\left|{\mathrm{y}}_1\hbox{--}\ {\mathrm{y}}_2\right|\right) $$

Although L_2_ is invariant under rotation, L_1_ will vary between the x-axis only and y-axis only difference as the point set **x** is rotated about the origin. Hence to examine the influence of distance bias, step 3 can be extended by considering a set of rotations **θ** between 0 and 90° using L_1_:

5. For each rotation θ_i_ ∈ **θ**
  - Rotate the original observed points **q** by θ_i_ and compute Ripley’s K using L_1_ distance.
  - Rotate the set of points **s** by θ_i_ about the origin to form the set **s’**.
  - For each distance threshold r_i_ count the number of points in **s’** within the Minkowski Distance L_1_ (Manhattan distance) d_i_ from each point in **s’**.

This metric is clearly justified for grid-like road patterns but may also be used when the geographic distance between points is difficult to define or involves some uncertainty.

## Case study: clustering of deliberate self-harm in an urban environment

This case study is based on data obtained from Invercargill, a small urban centre (population = 51,696 [16]) in the south of New Zealand. This was a retrospective 2-year audit based on file review of all patients who presented with DSH of any type to the Emergency Department or Emergency Psychiatric Service Team between January 2011 and Dec 2012. The audit was approved by the University of Otago Ethics Committee (H13/033). Data collected included demographic and clinical details and residential address.

**Fig. 1:**
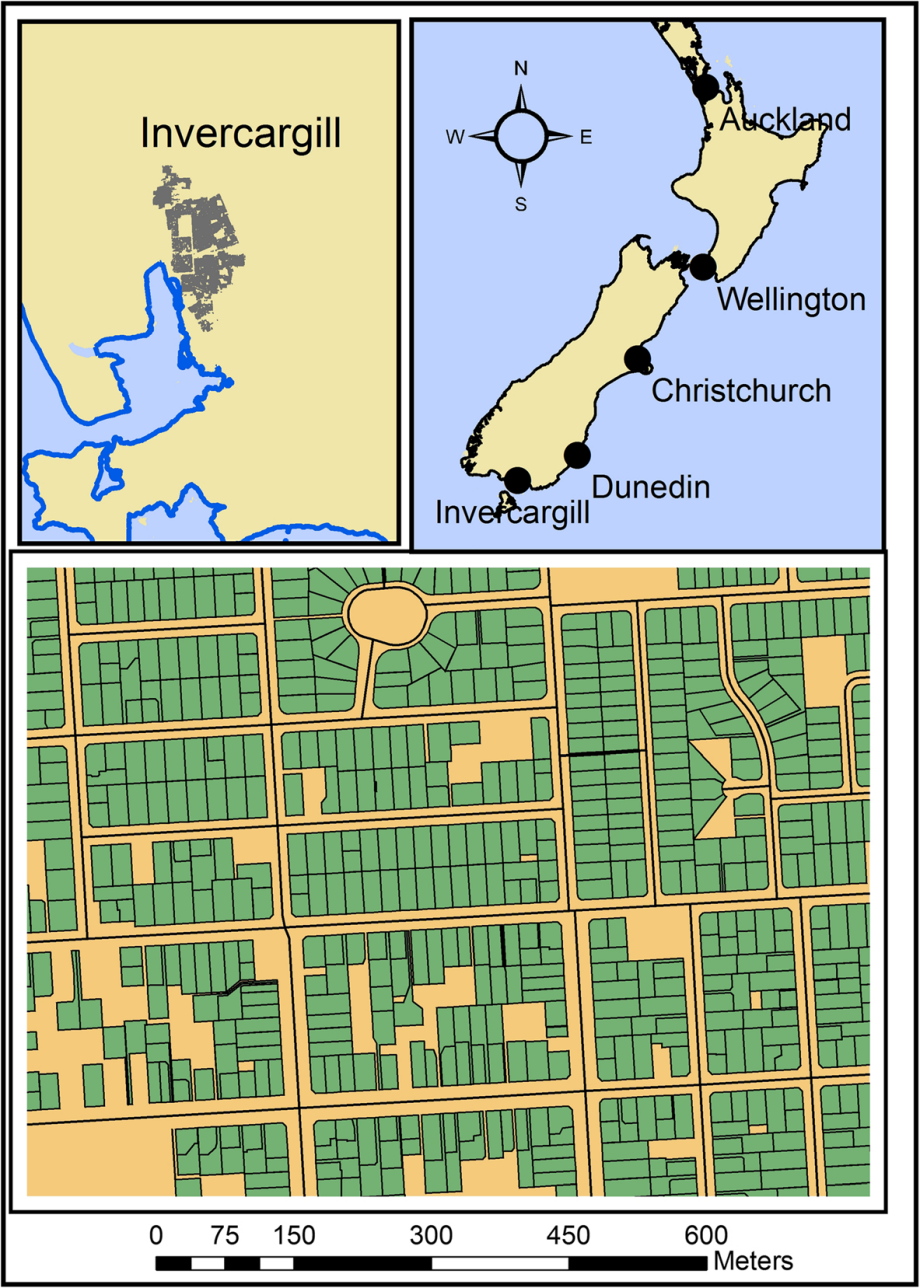
The urban area of Invercargill, New Zealand. *Lower panel* shows a portion of the residential land parcels (*green*) that represent the possible location of DSH cases. A simulated DSH episode can occur at the centroid of any residential parcel. Data obtained with permission from Land Information New Zealand (https://data.linz.govt.nz/)

Land parcels for Invercargill were obtained from the Land Information New Zealand online database [17]. The residential parcels were selected by selecting parcels where parcel_int = “DCDB” or “Fee Simple Title” AND statutory = NULL and survey_area >0. The parcels were then assessed by area, with the smallest 5 % and largest 5 % of parcels removed. This excluded schools, recreational areas and other parcels that were filling space but not accessible as polygons for residences. This was further reduced by using Google Maps to visually assess and remove those parcels that were shops or industrial areas. This resulted in 16,516 residential parcels that could be used as possible residential addresses. Note that every effort was made to reduce the number of residential parcels since this would reduce the likelihood of false clustering due to an oversized study area. Figure 1 shows the location of Invercargill (upper panels) and a section of the final residential parcels used in this study (lower panel).

The initial individual DSH data (*n* = 291, of which there were 245 unique individuals) was reduced to those that intersected the residential parcels (*n* = 164 with 134 unique individuals; data that were not included were for individuals who lived outside of the urban boundaries). Since we were interested in evidence for clustering and social contagion, only index episodes for a given location were kept. This meant that individuals with repeat DSH at the same address were removed; however the same individual who repeated DSH at different addresses, or a different individual at the same address, were kept in the dataset. The final DSH data consisted of 136 index episodes, with two repeat individuals. A measure of socio-economic quality of life, the New Zealand Deprivation Index (NZDep) was obtained based on the New Zealand Census data of 2006. NZDep is based on proportional measures of nine variables and constructed as a weighted sum determined by a principal component analysis of variable importance [18]. Deprivation index is a small area measure ranging from 1 (high quality) to 10 (poorest).

**Fig. 2:**
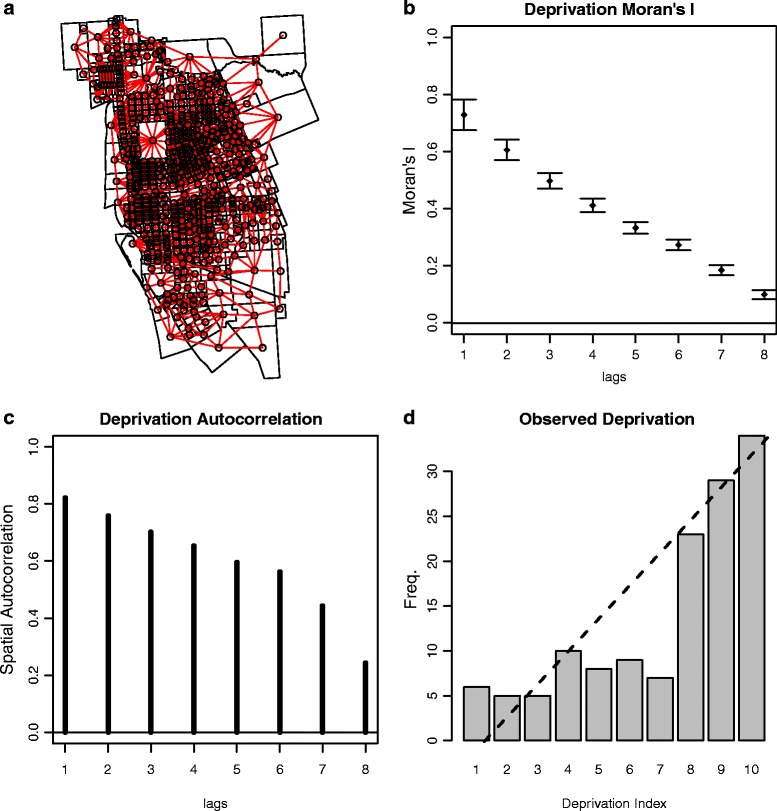
Panel (**a**) shows the small area meshblocks for urban Invercargill. The network represents the nearest neighbour connections used for spatial correlation. Clustering of New Zealand Deprivation Index is shown in panels (**b**) and (**c**). Moran’s I and the autocorrelation coefficient [19] are shown for increasing lag (steps) from any meshblock. The network model is used to determine the nearest neighbour (lag 1), 2nd nearest neighbour (lag 2), etc. Both measures show significant clustering of deprivation for several neighbourhood steps. The associated frequency of DSH index episodes and deprivation is shown in panel (**d**). The linear model (*dashed line*) has an adjusted R^2^ = 0.69

Figure 2 shows the meshblocks for the Invercargill urban area, and the spatial Moran’s I and autocorrelation measures [19] for the deprivation index associated with each meshblock (Panels A, B and C). Since observed DSH episodes are not uniform across deprivation (Panel D), spatial clustering of DSH will be observed due to the underlying clustered social structure of the urban environment. A similar relationship has been previously observed for suicide [14] and in a DSH young cohort study in New Zealand [20].

Although previous work on attempted suicide in New Zealand [10] suggested the existence of social contagion for space-time patterns, no account was made for the inherent clustering of social structure. Normally social structure is accounted for through incorporating their description into a regression model (see for example [11, 13]) however the use of a distance-based metric for clustering has no formal model for this type of integration. Hence a Monte Carlo simulation is appropriate for determining the null hypothesis [21], while removing the social clustering of deprivation as a model for DSH.

**Fig. 3:**
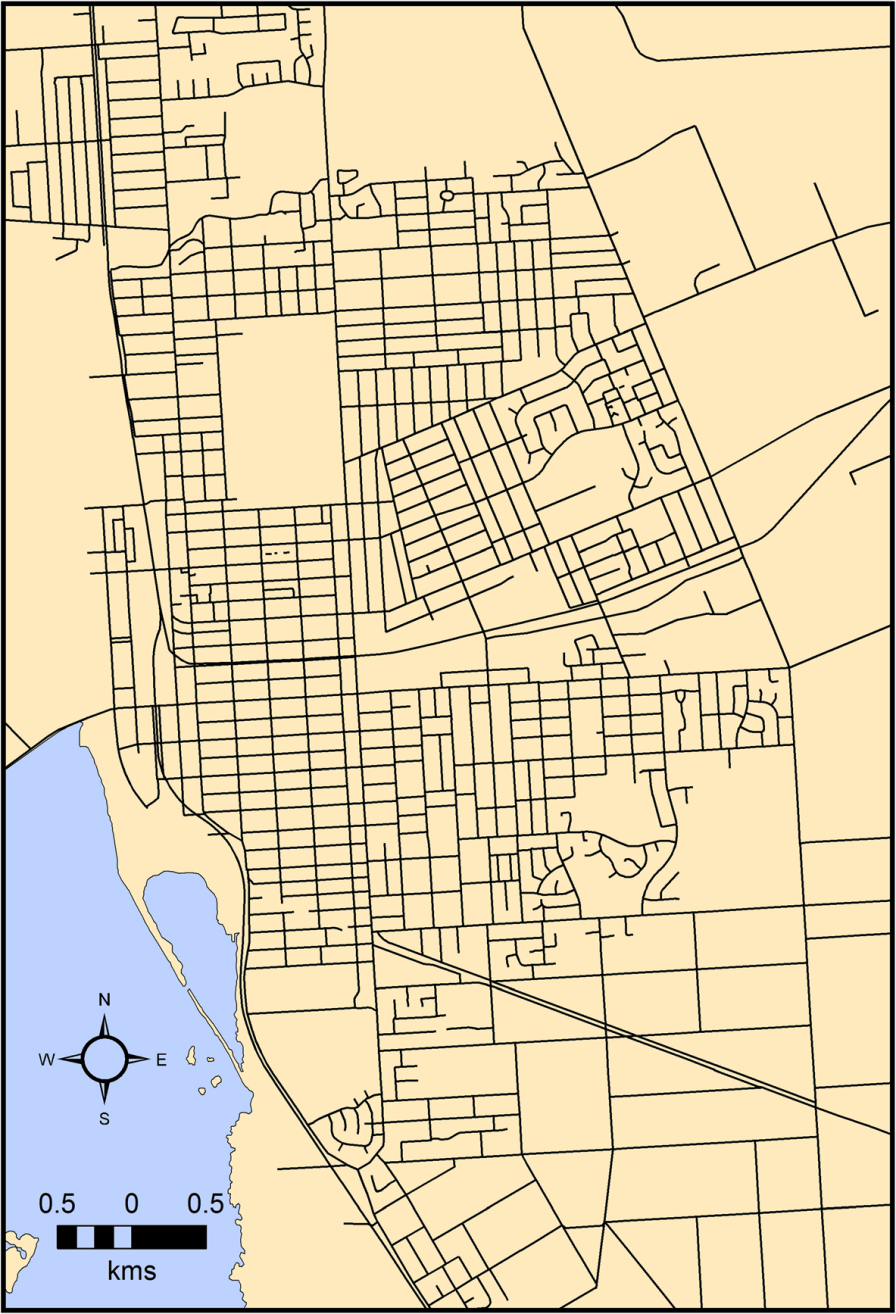
The road pattern for urban Invercargill is largely based on a grid (data obtained with permission from Land Information New Zealand (https://data.linz.govt.nz/) Rotations of L_1_ will capture an approximate network road distance and the orientations of road sections

For the Invercargill DSH data, the set **x** corresponds to the centroids of each residential parcel, the set **y** corresponds to the observed index events, and the marks M = {1…10} are the deprivation index. The grid-like pattern of roads within urban Invercargill (Fig. 3) justifies the use of rotated L_1_ distance measures to reducing the bias with Euclidean distance and increase confidence in any observed clustering of DSH.

## Results

**Fig. 4:**
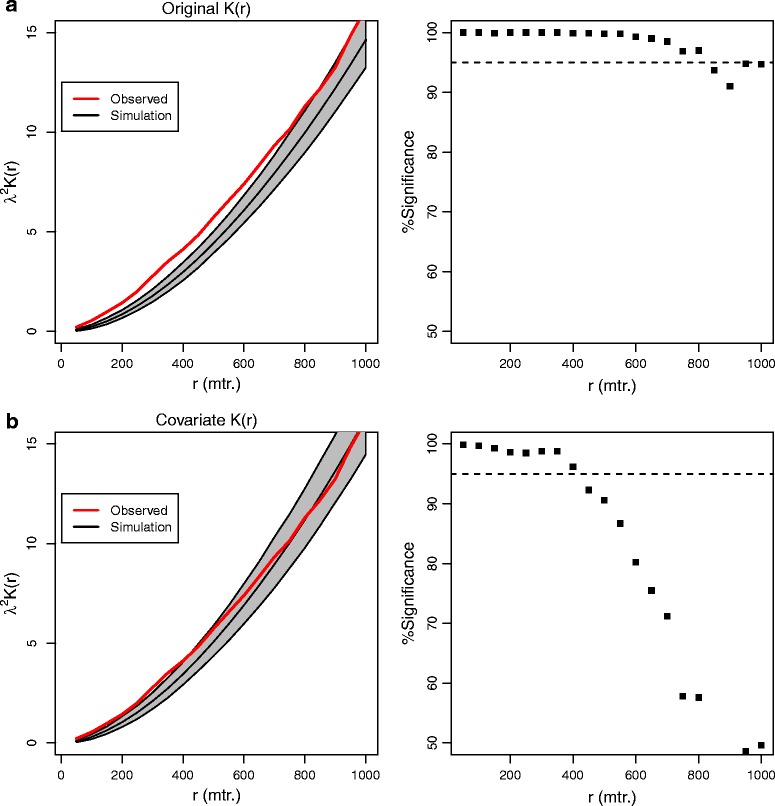
K(r) using Euclidean distance (L_2_). Panel (**a**) (*left*) shows K(r) when the social structure of deprivation is not taken into account. Panel (**a**) (*right*) shows the significance level above the median envelope K(r) value for clustering occurs up to ~800 m. The dashed line shows the one-sided 95 % confidence interval. Panel (**b**) (*left*) shows K(r) after clustering due to deprivation is removed. Panel (**b**) (*right*) shows that significant evidence for clustering reduces to ~500 m

Figure 4 shows K(r) estimated with uniform mark distribution (Panel A) and when the covariate distribution of deprivation index is taken into account (Panel B). Note that the y-axis is calculated as λ^2^K(r) which gives the expected number of points within r of an observed point. Panel A shows that without accounting for social structure (deprivation) clustering of index episodes is significant for all distances up to ~800 m. However, Panel B evidence for clustering is only apparent up to ~500 m once deprivation is accounted for when estimating K(r).

**Fig. 5:**
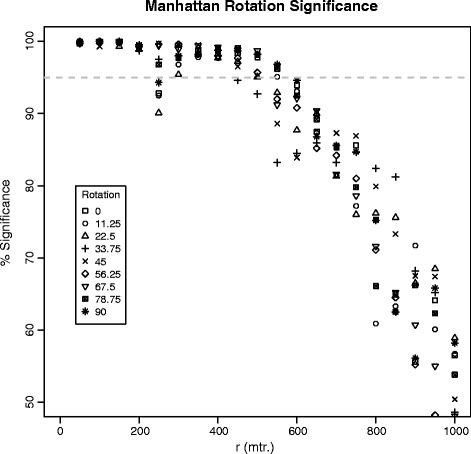
One-sided significance measures for K(r) using rotation of observed and simulated events with Manhattan distance. The dashed line indicates a one-sided 95 % significance level

Figure 5 shows a range of K(r, θ) values using L_1_ distance. Although some rotations (such as 22.5°) were below the 5 % threshold of evidence for clustering, it is apparent that for almost all rotations, clustering was significant up to ~500 m.

## Discussion

The original formulation of the second-order estimate for clustering K(r) assumes a stationary process generating the intensity of observed points and no constraint regarding the placement of points in the study area. However, there are many situations where the possible observation of a point is space is constrained due to the nature of the observed process, or through explicit constraints in the way that the defining space is created. For example, a residential analysis of patterns assumes that people live at valid addresses that do not include parks, businesses, etc., while an analysis of road accident clustering is constrained to locations on a road network. The use of individual data for health analysis will increase with improved data collection and the linked integration of datasets. The method presented here addresses some aspects of how to consider spatial clustering when individual data is used within a constrained spatial region and where a clustered covariate relationship exists. The results for DSH clustering, as shown in Fig. 4, show that once social structure is accounted for there still exists evidence for clustering up to ~500 m. This clustering may suggest aspects of social contagion [11], especially given evidence for clustering is demonstrated with the rotated L_1_ metric.

The issue of clustering and a distance metric is a difficult concept to manage and quantify with the increased use of social media as a tool for communication. Physically being close is no longer a requirement for proximity and social influence [22]. However since social networking tools are independent of location, DSH that derives from these influences should be spatially random once clustered covariates are managed.

The evidence for clustering presented in Fig. 5 suggests that there is a physical (geographic) relationship between individuals and DSH, although the study here has a number of limitations. The dataset is restricted to just 2 years of observations, and for only a single community. Both of these aspects limit any generalisation but do suggest that further work extending the data collection period and range of urban settings would be useful. In addition, the clustering method assumed a single spatial covariate (deprivation), however there could be other clustered covariates such as alcohol outlets [23, 24] that are creating the observed pattern for DSH clustering. This problem can be handled by extending the marked point pattern probability method to incorporate a multivariate density analysis [25] to create a probability surface for selecting fixed locations. Given that many physical constructions, such as alcohol outlets, are also often correlated with deprivation [23] may mean that handling a single variable that captures socio-economic structure is sufficient for estimating DSH clustering. Further work is required to determine the impact on clustering estimation with other configurations in the urban environment.

The concept of stationarity in space and time did not need to be considered here given the short time-frame and small urban area. However, although a longer time period and/or larger urban centre would produce a greater number of cases this would also increase the possibility of non-stationarity in the clustering behaviour. This would require additional methods for both detection and handling. Concepts such as non-stationarity in space and time are difficult to manage when assessing clustering and a likely solution would be to treat the clustering algorithm as a set of local statistics [26]. This is clearly future work but should be considered when large areas or long time frames are used in any assessment of spatial patterning.

Finally, extensions to Ripley’s K include a cross K function [5], which examines the relationship between two sets of finite (but differently marked) point observations. Extensions of the finite method to a cross function would allow questions of clustering to be related to point data that was not associated with the attributes of individuals and therefore extend the possible applications within the health domain.

## Conclusions

Point-based analysis is normally considered for a planar space with no placement constraints. In addition, since health-related data are often correlated with other social patterns that may have spatial structure (e.g. deprivation), there is a requirement to take these into account to handle bias in estimating clustering at a range of scales. The finite-location method presented here is simple to implement and allows any point-based health-related problem to be assessed for clustering. In addition, the use of a rotated L_1_ distance metric allows a more rigorous assessment of the observed clustering effect by determining the influence of the assumption of Euclidean distance when assessing K(r). This paper supports previous work on the influence of social deprivation on clustering of DSH in a small urban centre (8). In addition, evidence for social contagion has been demonstrated for DSH at small distance scales.

The presented finite point Ripley’s K approach allows an assessment of point-based observations, while handling a spatially clustered covariate and addressing distance bias. This paper is the first work to demonstrate social contagion as a likely influence for DSH at small distance scales within an urban centre. Whether this relationship can be generalised across different communities will require further studies of DSH in other urban environments. In addition, the relationship between covariates, clustering and health measures needs to be examined in more detail. It will therefore be important to confirm the utility of this approach in other urban settings using different outcome measures and covariates.

## Abbreviations

CI: Confidence interval
CSR: Complete spatial randomness
DSH: Deliberate self-harm
NZDep: New Zealand Deprivation Index
